# A nontrivial differential diagnosis in COVID-19 pandemic: a case report and literary review of amiodarone induced interstitial pneumonia

**DOI:** 10.2217/fca-2020-0168

**Published:** 2020-12-17

**Authors:** Luigi Cappannoli, Alessandro Telesca, Roberto Scacciavillani, Edoardo Petrolati, Andrea Smargiassi, Alessia Rabini, Massimo Massetti, Filippo Crea, Nadia Aspromonte

**Affiliations:** ^1^Catholic University of Sacred Heart, Rome, Italy; ^2^Department of Cardiovascular & Thoracic Sciences, Fondazione Policlinico Universitario A. Gemelli IRCCS, Rome, Italy

**Keywords:** amiodarone, coronavirus, COVID-19, differential diagnosis, drug toxicity, interstitial pneumonia, organizing pneumonia

## Abstract

Amiodarone is a drug commonly used to treat and prevent cardiac arrhythmias, but it is often associated with several adverse effects, the most serious of which is pulmonary toxicity. A 79-year-old man presented with respiratory failure due to interstitial pneumonia during coronavirus disease 2019 (COVID-19) pandemic. The viral etiology was nevertheless excluded by repeated nasopharyngeal swabs and serological tests and the final diagnosis was amiodarone induced organizing pneumonia. The clinical and computed tomography findings improved after amiodarone interruption and steroid therapy. Even during a pandemic, differential diagnosis should always be considered and pulmonary toxicity has to be taken into account in any patient taking amiodarone and who has new respiratory symptoms.

## Lay abstract

Amiodarone is a drug used to treat and prevent heart rhythm alterations (cardiac arrhythmias). Although it is effective, it is associated with some side effects, including pulmonary toxicity (in 2–18% of patients), which is a serious side effect impacting the lungs of the patient. A 79-year-old man presented with a dry cough and shortness of breath during the coronavirus disease 2019 (COVID-19) pandemic, but repeatedly tested negative for the virus. The diagnosis was determined to be amiodarone induced pneumonia, a condition that inflames the air sacs in the lungs. The patient stopped taking amiodarone and steroid therapy was started, leading to an improvement in the patient's condition. Even if the diagnosis initially seemed to be of COVID-19, amiodarone toxicity should always be considered in patients taking this drug and with respiratory symptoms.

Amiodarone is a bi-iodinated benzofuran derivative class III antiarrhythmic agent (according to Vaughan–Williams classification) [1] used to treat and prevent several cardiac arrhythmias, both supraventricular and ventricular. Amiodarone and its main metabolite mono-N-des-etil-amiodarone have a long half-life (55–60 days) and high lipid solubility, thus accumulating largely in adipose tissue and highly perfused organs, such as liver, lungs and spleen [2–5]. Amiodarone is a very common use drug, but it is frequently associated with several adverse effects, including bradycardia or atrioventricular (AV) blocks, hypothyroidism or hyperthyroidism, blue–grey skin discoloration and photosensitivity, elevated liver enzymes (ALT or AST higher than two-times normal values), corneal microdeposits, anorexia and nausea. Opthalmological evaluation, a yearly ECG and semi-annually thyroid and liver profiles are therefore useful in follow-up. However, the most serious adverse effect is amiodarone pulmonary toxicity (APT) [6], a potentially limiting factor for its use, frequently misdiagnosed, which ranges from acute/subacute interstitial pneumonias, organizing pneumonia (OP), acute respiratory distress syndrome (ARDS), diffuse alveolar hemorrhage, pulmonary nodules/masses and pleural effusion. An accurate differential diagnosis is therefore mandatory. The incidence of APT is 4–17% [7] and risk factors include dosage and duration of therapy (even if a real‘threshold’ does not exist), increased patient age (threefold for every 10 years in patients over 60 years), male sex, preexisting lung disease, underling pathologies, oxygen administration and invasive or surgical procedures, primarily thoracic ones [8–12]. angiotensin converting enzyme inhibitors-inhibitors and angiotensin receptor blockers seem to be associated with a lower incidence of APT: they increase isoform 2 of ACE expression and activity, which degrades Angiotensin II to Ang1–7, hence diminishing Angiotensin II receptor 1-mediated deleterious effects of enhancing amiodarone-induced apoptosis of alveolar epithelial cell, that in turn plays a central role in the development of acute lung injury [13–15].

## Case presentation

We present the case of a 79-year-old man suffering from chronic HF with reduced ejection fraction in postischemic dilated cardiomyopathy, previously implanted with implantable cardioverter-defibrillator in secondary prevention, affected by paroxysmal atrial fibrillation and ascending aortic aneurysm (55 mm), with nonrelevant previous pulmonary history, never smoker, without occupational exposure. Dyspnea, dry cough and signs of respiratory failure without fever appeared at the end of February 2020 and he was hospitalized at the beginning of March 2020.

**Figure 1.**
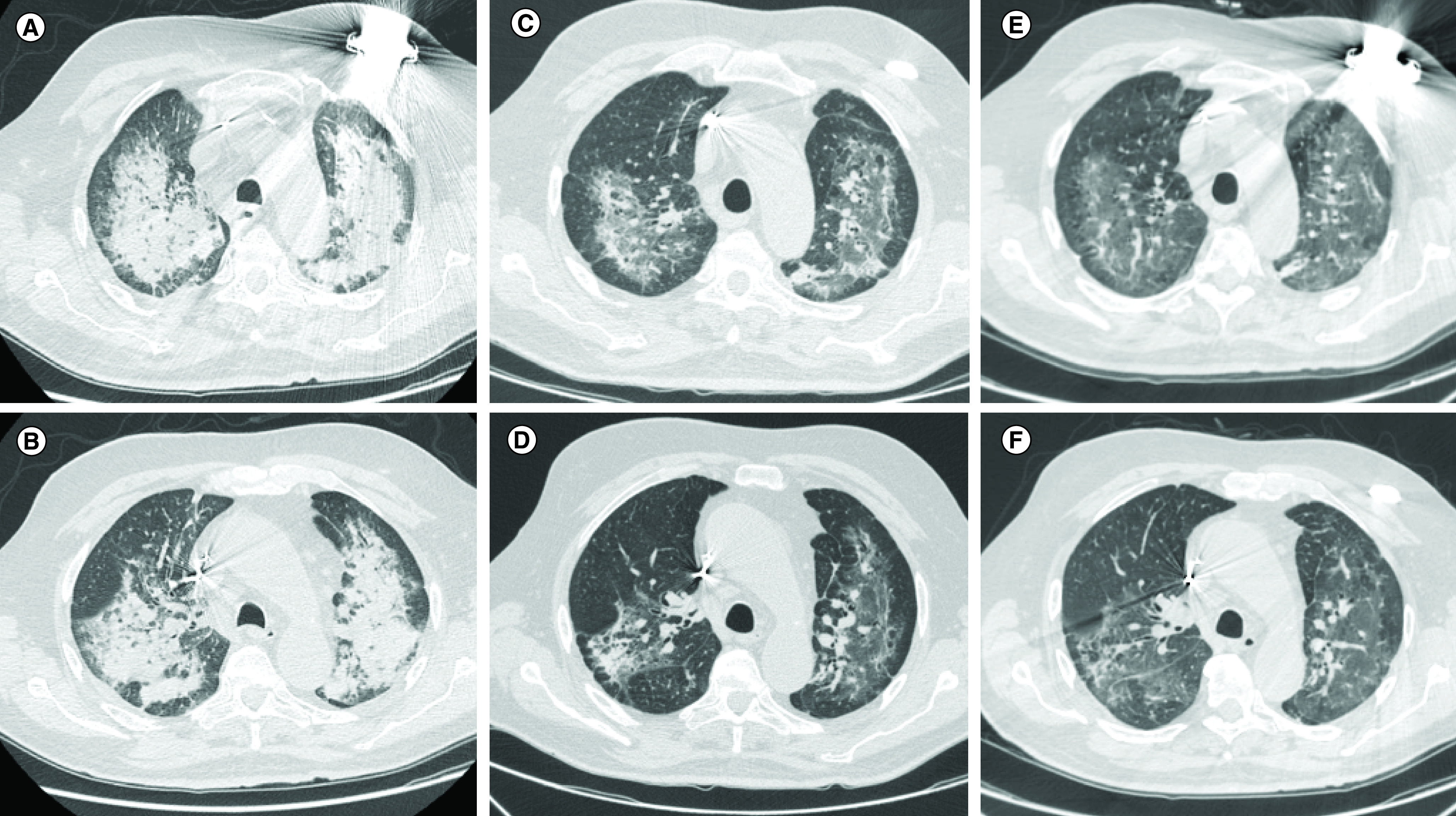
High-resolution computed tomography of a 79-year-old man with amiodarone induced organizing pneumonia. Extended multifocal parenchymal thickening at the **(A)** apical and **(B)** lower lobes, bilaterally, with vast ground glass areas and pseudonodular parenchymal consolidations. Progressive resolution of organizing pneumonia after 2 months **(C & D)** and after 3 months of steroid therapy and drug interruption **(E & F)**, with persisting ‘ground glass’ areas associated with fibrotic-cicatricial manifestations, such as retractions of costal pleural sheets, mostly in lower lobes.

The patient’s home therapy was pantoprazole 40 mg daily, atorvastatin 20 mg daily, amiodarone 200 mg daily, bisoprolole 3.75 mg, furosemide 25 mg twice a day and apixaban 2.5 mg twice a day (eGFR 38 ml/min) at admission. The initial laboratory examination revealed a normal white blood cells (WBC) count (6.74 × 10^9^/l) with a normal neutrophilic and lymphocyte ratio and increased creatinine value (2.16 mg/dl). A first chest high-resolution computed tomography (HRCT) scan (Figure 1A & B) documented vast areas of bilateral parenchymal consolidation and ground glass opacities (GGO) in the upper lung lobes (Figure 1A), with prevalent perihilar distribution in the lower lobes with air bronchiologram (Figure 1B). These findings were compatible with interstitial pneumonia, in particular OP. CT also showed enlargement of mediastinal lymph nodes (paratracheal and precarenal ones) and pleural effusion, mostly on the left.

The differential diagnosis was challenging, and it included: coronavirus disease 2019 (COVID-19) pneumonia; cardiogenic pulmonary oedema; viral, bacterial and autoimmune pneumonia; APT. In the high suspicion of COVID-19-related pneumonia, two nasopharyngeal swabs for SARS-CoV-2 were performed (at admission and 48 h later) which resulted negative. The occurrence of two consecutive false negative results was considered highly unlikely and, moreover, serological tests for SARS-CoV-2 1 month after discharge were also negative for both IgM and IgG, confirming the exclusion of COVID etiology. There were no clinical and instrumental signs of acute heart failure (HF). Peripheral edema or ascites were absent. NT-proBNP plasmatic concentration was not elevated in comparison with patient’s baseline value. Transthoracic echocardiogram confirmed postischemic dilated cardiomyopathy with a reduced ejection fraction (32%), unchanged from the previous control. Moreover, HRCT findings were not typical of HF (see ‘discussion’). Therefore, a cardiogenic pulmonary edema was excluded. To rule out other causes of interstitial pneumonia associated with respiratory failure, a large number of laboratory tests were performed, such as plasma level of beta-D-glucan, anti-ENA SSB/La, SSA/Ro, Sm, RNP antibodies, viral serologies and bacterial research in sputum culture. They all resulted negative.

HRCT findings and the exclusion of alternative diagnosis therefore raised the suspicion of amiodarone induced OP. Amiodarone was in fact started 8 months prior to hospital admission with intravenous load, followed by oral administration of 200 mg three-times a day, gradually deescalated to a dosage of 200 mg daily after 8 weeks. Amiodarone was therefore immediately suspended and steroid therapy (prednisone 40 mg/day) was started, with clinical improvement. The CT scans at follow-up in May (Figure 1C & D) and June (Figure 1E & F) showed an absorption stage with a partial resolution of OP characterized by progressive reduction of the parenchymal consolidations of the upper lobes, with persisting ‘ground glass’ areas, and with slight signs of retraction on the pleural sheets and bronchovascular structures. Pleural effusion was absent bilaterally. Signs and symptoms of respiratory insufficiency further improved.

## Discussion

Amiodarone is a drug largely used by cardiologists for its efficacy in preventing and treating supraventricular and ventricular arrhythmias. Nevertheless, it is associated with a variety of side effects, including pulmonary toxicity. There are two different categories of pulmonary involvement following amiodarone assumption: asymptomatic lipid pneumonia and APT. In turn APT can be caused by two possible mechanisms: a direct cytotoxic effect or an immuno-mediated mechanism, supported by immunologic markers in the blood stream and lungs of patients and CD8^+^ lymphocytosis in bronchoalveolar lavage (BAL), with imbalance between T-helper type I and II subpopulations and cytokines [16,17]. APT is less common than thyroid, eye and skin toxicity, but it is the most dangerous one because it may occur as a subacute/chronic onsetting alveolar or interstitial pneumonia with vary degrees of fibrosis, as well as an acute respiratory distress with severe hypoxemia [18]. High cumulative dose and duration of therapy exceeding 2 months, together with pre-existing lung disease, are important risk factors of APT. It affects about 6% of patients receiving a daily dose of 400 mg (or more) over 2 or more months, with a mortality rate of 10–20% [19]. It is characterized by insidious onset of non-productive cough and/or progressive dyspnea on exertion, usually within 6–12 months from starting amiodarone, but it can occur at any time after the treatment is initiated [20]. Low-grade fever or pleuritic chest pain are rarely present. The worst manifestation of APT is a rapidly progressing diffuse pneumonitis with acute respiratory failure and a typical panel of ARDS. In 5–7% of patients amiodarone pneumonitis is followed by amiodaron-induced pulmonary fibrosis, irreversible and with a poor prognosis. Alveolar hemorrhage and hemoptysis are possible, but unusual [21]. On laboratory data, leukocytosis is often present, rarely due to eosinophilia [22], there could be also a nonspecific elevation of lactic dehydrogenase or serum IL-6, a mucin like glycoprotein expressed on type II pneumocytes and bronchiolar cells. Pulmonary function tests usually show a restrictive syndrome with decreased forced vital and total lung capacities and a reduction in diffusing capacity of the lungs for carbon monoxide more than 15–20% [23]. Pulmonary imaging is essential for the diagnosis and it is characterized by the presence on HRCT of extensive and severe bilateral patchy GGO with honeycombing, localized or diffuse, mono or bilateral, parenchymal (interstitial or alveolar) infiltrates, high attenuation consolidations, also called ‘amiodaronoma’, especially in the right upper lobe [24]. High attenuation, associated with the iodinated properties of the drug, may also appear in the liver and spleen and evidences suggest that lesions >70 Hounsfield Units (HU) may be related to amiodaron toxicity [25]. On microscopic inspection on BAL or transbronchial biopsy, a characteristic finding is the presence of lipid-laden foamy macrophages in alveolar spaces, even if not specific because they are also present in nontoxic patients receiving amiodarone, usually associated to hyperplasia of type II pneumocytes and widening of alveolar septae with a cellular inflammatory infiltrate and varying degrees of interstitial fibrosis [26]. Open lung biopsy should be avoided because APT may worsen after thoracic surgery. Exclusion diagnosis include lung viral or bacterial infection, HF, exogenous lipid pneumonia, bronchoalveolar carcinoma and lymphoma. The disease usually responds to drug discontinuation and corticosteroid administration within a period of 1–6 months. Recurrences are more frequent with steroid tapering in patients with excess adipose tissue [27].

APT is therefore prevalently an exclusion diagnosis, so that a number of alternative conditions have always to be taken into account before considering it.

### COVID-19 pneumonia

In the COVID-19 era, the presence of dyspnea and respiratory impairment in patients with interstitial involvement on chest imaging makes it mandatory to suspect a COVID-19 pneumonia, that represents the most common manifestation of the disease. The most diffuse clinical manifestations are fever (>38°C in most cases), dyspnea, dry cough or expectoration with or without rhinorrhea, hypo-anosmia and/or ageusia, fatigue, headache, diarrhea and myalgia up to more severe conditions such as ARDS and respiratory failure, which sometimes require advanced respiratory assistance [28–30]. COVID-19 first affects the terminal bronchioles and surrounding parenchyma, and then develops into infiltration of pulmonary lobules and lastly diffuse alveolar damage [31]. Evidences also suggest a predisposition to thrombotic and thromboembolic disease in these patients [32,33], that were excluded in our case. Lab values in most of COVID-19 patients show normal or low WBC count, elevated neutrophil ratio, serum C-reactive protein, procalcitonin and lactic dehydrogenase and decreased lymphocyte ratio and lymphocyte count. The standard diagnosis of COVID-19 infection requires the identification of viral RNA by the real-time reverse transcriptase PCR essay of respiratory secretions obtained by nasopharyngeal and/or oropharyngeal swab, BAL or tracheal aspirate, with a sensitivity of 32–71% [34–36]. In case of negative result and if persisting high clinical suspicion of COVID-19, it is advised to perform chest CT, that has high sensitivity (75–94%) despite a reduced specificity [37] and shows GGO with bilateral (most) peripheral involvement in multiple lobes progressing to crazy paving pattern, fine reticular opacity and vascular thickening inside the lesions [30,38]. These radiological findings usually present with bilateral and multilobar distribution and a predominant involvement of subpleural/peripheral and posterior lung parenchyma [39], particularly in the lower lobes [40]. Several days after the onset of disease, in most patients linear consolidations and areas of GGO surrounded by peripheral consolidation (reverse halo sign) appear, suggesting OP. Uncommon HCRT features are multifocal nodular appearance with irregular margins, enlargement of mediastinal lymph nodes, pleural effusion and bronchial wall thickening, related to severe disease [38,41]. The most serious pathological pattern of the pulmonary damage caused by SARS-CoV-2 is a condition of acute lung injury, with a wide spectrum of histological pattern ranging from diffuse alveolar damage with hyaline membrane formation to OP [42–44].

### Alternative diagnosis

Cardiogenic pulmonary edema is a very common cause of diffuse GGO on HRCT. Typical HRCT features in these patients are the enlargement of the pulmonary veins and smooth thickening of the interlobular septa and peribronchovascular bundles, that were absent in our patient. Besides, HF usually presents a central predominance with sparing of the peripheral portions of the lungs [45], that instead were involved in the presented case. At last, in cardiogenic pulmonary edema the lung lesions can be significantly improved after effective anti-HF treatment. Table 1 summarizes clinical features, radiological findings and laboratory characteristics of APT, COVID-19 pneumonia and cardiogenic pulmonary edema to guide differential diagnosis. Viral, bacterial and autoimmune pneumonias can be usually easily ruled out with laboratory tests including WBC count, specific antibodies search, beta-D-glucan, viral serologies (and in some cases search for the viral genome in blood samples) and bacterial search in sputum culture.

**Table 1.** Clinical features, radiological findings and laboratory characteristics of amiodarone pulmonary toxicity, COVID-19 pneumonia and cardiogenic pulmonary edema to guide differential diagnosis.

	Amiodarone pulmonary toxicity	COVID-19 pneumonia	Cardiogenic pulmonary edema
Clinical features	Dyspnea Anorexia Dry cough Hypoxemia	Fever Cough Anosmia and ageusia Shortness of breath Diarrhea and myalgia	Orthopnea Extreme shortness of breath Wheezing or gasping for breath Wheezing Swelling in lower extremities
Radiological findings	GGO Peripheral involvement (mainly) Progressive fibrosis	GGO Bilateral peripheral involvement Fine reticular opacity and vascular thickening inside the lesions Progressive acute lung injury (hyaline membrane formation up to OP)	GGO Central predominance with sparing of the peripheral portions of the lungs Peribronchovascular bundles Pleural effusion
Laboratory/instrumental characteristics	Leukocytosis (rarely due to eosinophilia) Restrictive pattern on spirometry	Lymphopenia Elevated inflammatory markers (CRP; IL-6) Elevated LDH and D-dimer	NT-proBNP elevation EF reduction on echocardiogram

COVID-19: Coronavirus disease 2019; CRP: C reactive protein; EF: Ejection fraction; GGO: Ground glass opacities; LDH: Lactic dehydrogenase.

## Conclusion

Pulmonary toxicity is relatively frequent and occurs in 2–18% of patients receiving amiodarone, usually during long-term therapy with high cumulative doses, and can lead up to lung fibrosis and fatal respiratory failure [6]. The most common CT findings include septal thickening and interstitial pneumonia which can result in OP. The differential diagnosis of APT is mandatory, but can however be challenging, especially in COVID-19 era when interstitial pneumonias are easily attributed to SARS-CoV-2 infection. A temporal relationship of amiodarone intake for months or years could therefore be a key point in the differential diagnosis, as well as the negativity of swabs and serology for viral infection. Moreover, clinical and radiological presentation of interstitial pneumonia can also be similar to those of HF: pulmonary edema causes shortness of breath and CT ground-glass opacities and thickening of interlobular septum, but with prevalent central distribution and higher expansion of small pulmonary veins. When amiodarone therapy is begun it is mandatory to perform a basal and yearly chest x-ray and pulmonary function tests, including a diffusing capacity of the lungs for carbon monoxide. In this context, it is important to investigate when symptoms began or any recent changes in therapy and APT should always be suspected in any patient taking amiodarone who has new or worsening symptoms and/or new infiltrates on chest x-ray or CT scan.

**Summary points**

- The presence of dyspnea, other respiratory symptoms and ground glass opacities on chest high-resolution computed tomography requires a challenging differential diagnosis, especially in COVID-19 era.
- The main adverse effects associated to amiodarone involve the lungs, thyroid, eye, liver and skin.
- There are two kind of pulmonary involvement due to amiodarone assumption: an asymptomatic lipoid pneumonia and amiodarone pulmonary toxicity (APT), with an incidence that ranges from 4 to 17% and a mortality rate of 10–20%.
- Risk factors for APT include dosage and duration of therapy, patient age, male sex, pre-existing lung disease, oxygen administration and invasive or surgical procedures.
- The worst complication of APT is a rapidly progressing diffuse pneumonitis with acute respiratory failure and acute respiratory distress syndrome, followed in 5–7% of patients by pulmonary fibrosis, only partially reversible and with a poor prognosis.
- On high-resolution computed tomography, APT is characterized by severe bilateral patchy ground glass opacities with honeycombing, localized or diffuse, mono or bilateral, parenchymal infiltrates, high attenuation consolidations, especially in the right upper lobe.
- APT is a diagnosis of exclusion, after considering viral or bacterial pneumonias, cardiogenic pulmonary edema, exogenous lipid pneumonia, bronchoalveolar carcinoma and lymphoma.
- Clinical status APT-patients usually improves after drug discontinuation and corticosteroid administration within a period of 1–6 months.
